# Illumination Sensor for Reflection-Based Characterisation of Technical Surfaces

**DOI:** 10.3390/s26041256

**Published:** 2026-02-14

**Authors:** Tim Sliti, Nils F. Melchert, Philipp Middendorf, Kolja Hedrich, Eduard Reithmeier, Markus Kästner

**Affiliations:** Institute of Measurement and Automatic Control, Stiftung Gottfried Wilhelm Leibniz Universität Hannover, An der Universität 1, D-30823 Garbsen, Germany

**Keywords:** BRDF, reflectance, surface measurement, roughness

## Abstract

The condition of technical surfaces strongly influences the functionality and lifetime of many components. In particular, the performance of aero-engines can be impaired by increased roughness of the turbine blade surfaces. In this work, an LED- and camera-based illumination sensor is presented for reflection-based characterisation of turbine blade surfaces, with a focus on rapid, wide-area assessment rather than direct roughness measurement. Traditional roughness measurements (e.g., profilometry, confocal microscopy) provide micrometre-scale height information but are limited in working distance and measurement volume, making complete surface coverage time-consuming. The proposed sensor acquires multi-illumination image data, from which an anisotropic BRDF (bidirectional reflectance distribution function) model is fitted on a per-pixel basis to obtain reflectance parameters. Independently, surface roughness parameters (Sa, Sq, Sz, Ssk, Sku) are measured using a confocal laser scanning microscope in accordance with ISO 25178 and used as reference data. Using two turbine blades with contrasting surface conditions (comparatively smooth vs. visibly rough), the study qualitatively investigates whether there are indications of relationships between BRDF model parameters and roughness characteristics. The results show weak relationships with height-based parameters (Sa, Sq, Sz), but clearer trends for distribution parameters (Ssk, Sku) and a good qualitative agreement between directional BRDF parameters and texture orientation. These findings indicate that the illumination sensor provides a complementary, reflectance-based approach for surface condition triage in MRO and QA contexts, highlighting regions that warrant more detailed roughness measurements. Extension of the approach to other component geometries and a comprehensive quantitative analysis of BRDF–roughness relationships are planned for follow-up studies.

## 1. Introduction

Assessing the condition of a component is an important step, e.g., in maintenance, repair and overhaul (MRO) as well as in quality assurance (QA). Here, different parameters of the component can be acquired to ensure the functionality. Besides ensuring the dimensional accuracy, roughness parameters are particularly relevant for functional surfaces, as these have an influence on, for example, friction, wear or lubricity and can thus have a significant impact on the component’s properties. In the field of turbomachinery, Nardini et al. [1] were able to show that increased surface roughness influences the efficiency of aero-engines. The measurement of this roughness requires special measuring systems. These are usually limited in their working distance and space, which requires precise placement of the sample. Measuring larger areas is time-consuming, which is why single measurements are often only taken in relevant areas. Therefore it is sensible to detect these areas in advance.

In this work, we investigate if the task of distinguishing between aforementioned regions can be achieved with the developed illumination sensor with which the properties of technical surfaces are to be characterised. The illumination sensor uses an area-integrating, angle-resolved measurement method that aims at image-based, BRDF-controlled classification of the surface condition and can be classified according to the methods specified in ISO 25178-6 [2] (see Section 3.1). Using two turbine blades, it is evaluated whether there are indicators of relationships between roughness parameters measured using a confocal laser scanning microscope and BRDF model parameters, which can be acquired using the illumination sensor. For this purpose, the concept of reflection models, which are mainly used in computer graphics, is applied. These models are parameterised with model-specific parameters which often include a “roughness” parameter to model the diffuse reflection behaviour. To model direction-dependent phenomena, an anisotropic reflectance model is applied. The extent to which the various model parameters show relationships with measured roughnesses and their spatial orientation is investigated and discussed.

## 2. Materials and Methods

In the following sections, the different algorithms and concepts used in this work and related literature is presented. This includes the applied reflectance model, photometric stereo algorithms and various related strategies, which have been proposed to measure and fit a reflectance model to a dataset.

### 2.1. Reflectance Models

**Figure 1:**
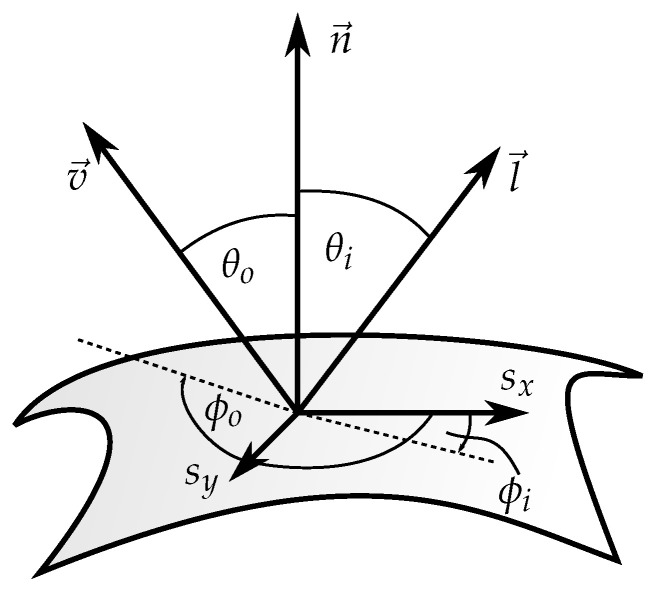
Reflection on a surface point with an incident beam ${\vec{l}}_{n}$, the outgoing beam $\vec{v}$ and the local surface coordinate system defined by $s_{x}$ and $s_{y}$ and $\vec{n}$.

Reflectance models are used to characterise the reflection properties of surfaces. A general model is the bidirectional reflectance distribution function (BRDF), which describes how each incident wavelength is reflected depending on its incident direction $\vec{l}$ and the direction of observation $\vec{v}$ (Figure 1) [3]. Taking the different vectors into account the BRDF can be formulated as

(1) $$f_{r}\left(\theta_{i},\phi_{i},\theta_{o},\phi_{o},\lambda \right).$$

where the suffixes *i* and *o* are respectively used for incoming and outgoing beams, and λ denotes the lights wavelength. Since this work does not distinguish between multiple wavelengths, λ will be left out in the future and all reflectance models are considered for the available spectrum.

Many different reflectance models have been developed to describe the interaction of light with surfaces. In [4], Guarnera et al. give an extensive overview over various BRDF models and implementations. These models are sorted by different properties like reciprocity, energy conservation or the ability to model anisotropic phenomena.

In this work, the anisotropic phong model introduced in [5] is used. Inspired by [6,7,8], this model obeys energy conservation and reciprocity laws while allowing modelling of anisotropic behaviour. The model is based on a diffuse ($f_{d}$) and a specular ($f_{s}$) portion

(2) $$f_{r}\left(\vec{l},\vec{v}\right)=f_{s}\left(\vec{l},\vec{v}\right)+f_{d}\left(\vec{l},\vec{v}\right)$$

and is controlled by four parameters:

- The specular component $R_{s}$.
- A diffuse reflectance component $R_{d}$.
- The directional parameters $n_{x}$, $n_{y}$ to shape the specular lobe.

The specular component $f_{s}$ is calculated with

(3) $$f_{s}\left(\vec{l},\vec{v}\right)=\frac{\sqrt{\left(n_{x}+1\right)\left(n_{y}+1\right)}}{8\pi }\frac{{\left(\vec{n}\;\cdot \;\vec{h}\right)}^{\frac{\left(n_{x}{\left(\vec{h}\;\cdot \;\vec{s_{x}}\right)}^{2}+n_{y}{\left(\vec{h}\;\cdot \;\vec{s_{y}}\right)}^{2}\right)}{\left(1-{\left(\vec{h}\;\cdot \;\vec{n}\right)}^{2}\right)}}}{\left(\vec{h}\;\cdot \;\vec{v}\right)\max\left(\left(\vec{n}\;\cdot \;\vec{l}\right),\left(\vec{n}\;\cdot \;\vec{v}\right)\right))}F\left(\left(\vec{v}\;\cdot \;\vec{h}\right)\right).$$

Here, $\vec{h}$ denotes the normalised half-vector between $\vec{l}$ and $\vec{v}$, $\vec{a}\;\cdot \;\vec{b}$ the dot product of $\vec{a}$ and $\vec{b}$, and *F* Schlick’s approximation of the Fresnel factor [7]:

(4) $$F\left(\left(\vec{v},\vec{h}\right)\right)=R_{s}+\left(1-R_{s}\right){\left(1-\left(\vec{v}\;\cdot \;\vec{h}\right)\right)}^{5}.$$

The diffuse portion of the model is

(5) $$f_{d}\left(\vec{l},\vec{v}\right)=\frac{28R_{d}}{23\pi }\left(1-R_{s}\right)\left(1-{\left(1-\frac{\left(\vec{n}\;\cdot \;\vec{l}\right)}{2}\right)}^{5}\right)\left(1-{\left(1-\frac{\left(\vec{n}\;\cdot \;\vec{v}\right)}{2}\right)}^{5}\right)$$

With these equations, it is possible to calculate the model-based intensity of a surface point given the model parameters and the normal, light and view vectors.

### 2.2. Photometric Stereo

Photometric stereo (PS) methods aim to estimate the surface normals of an observed 3D object, which is illuminated from varying light directions utilising one moving or multiple light sources. This work mainly applies the strategies introduced by Woodham [9]. Here, *N* images $I_{i}$ with varying light directions ${\vec{l}}_{i}\in R^{3\times 1}$ with i∈{0…N−1} are captured with a camera from a fixed view point. With given illumination strengths $r_{i}$, the following underdetermined equation system can be formulated for the relationship between intensities and surface orientation [10]:

(6) $$\left(\begin{array}{c} I_{0}\\ I_{1}\\ \vdots \\ I_{N-1} \end{array}\right)=\alpha \left(\begin{array}{c} r_{0}{\vec{l}}_{0}\\ r_{1}{\vec{l}}_{1}\\ \vdots \\ r_{N-1}{\vec{l}}_{N-1} \end{array}\right)\;\cdot \;\vec{n}=\alpha \;\cdot \;L\;\cdot \;\vec{n}.$$

Here, α is the surface albedo. For more than three images, L is overdetermined an the solution can be calculated by determining the pseudo inverse of L in a least squares manner.

This approach generally works well for lambertian surface reflection properties, but not for specular highlights. Since the results for the examined measurement objects were satisfying, more complex strategies have been foregone. An overview is given in [10,11] which summarise and compare different photometric stereo approaches. More recent work related to the surface characterisation of aero-engine components has been carried out by Ma et al. [12,13].

### 2.3. Measuring BRDF Data

Usually BRDF data are acquired with gonioreflectometers which are described by Hsia and Richmond in [14]. With this setup, it is possible to gather datasets which characterise the reflectance for different materials. The measurement is time-consuming and generates a large amount of data [4]. Other types of sensors have been developed utilising image-based measurements. Here, Marschner et al. [15] present a sensor to measure the isotropic BRDF of different materials including human skin by moving a handheld CCD camera with respect to a light source and capturing about 30 images. Each pixel is evaluated to cover the three-dimensional BRDF domain. The sample shape has to be analytically defined or known from 3D scanning. Matusik et al. [16] present a similar strategy using a stationary sample and camera with a moving light source and spherical specimen. There are many more setups like [17,18,19] which make use of mirror or LED setups to vary incident and outgoing angles. For a more complete summary of existing methods, refer to [4,20], since the aim of this work is not to measure BRDF, but rather to approximate the reflectance properties of surfaces. Another major difference is the specimen. While the previously mentioned works mainly use simple shapes like spherical, cylindrical or planar specimen with one material and thus only one BRDF per object, this work examines complex blade geometries with varying surface properties due to different signs of wear. Nevertheless, a lot of conclusions can be used and adapted for this work.

### 2.4. Fitting BRDF Models

While the datasets gathered with gonioreflectometers are generally large and can act as a look up table, they do not cover the whole domain of the BRDF [21]. Therefore it is desirable to represent the reflection properties in a functional form as provided by various BRDF models. As described in Section 2.1, these models are used to calculate the intensity based on the given surface normal, light and view vector and model-specific parameters. Since the input vectors are usually known from the measurement setup or calibrated beforehand, only the model specific parameters have to be estimated. In general, optimisation methods are used to determine these parameters by minimising some form of error metric. Kay and Caelli describe the fitting process using a nonlinear weighted least squares approach in [22] and address ill conditioning in the optimisation process. To improve the convergence, Yu et al. [23] describe a method which partially optimises the parameters in succession, rather than simultaneously. Another strategy is presented in [24], where an image-driven error metric is introduced to increase fidelity when fitting the models to real world data. Since this work will not make use of large datasets, the aim is not to find the best possible fit for the reflectance model or provide material specific model parameters, but to approximate surface properties on the measurement object. The applied fitting strategy is presented in Section 3.2.3.

## 3. Experimental Setup

To gather information about the measurement object, a sensor had to be designed. The concept and implementation of this sensor is presented in Section 3.1. The data acquired with the proposed system is further evaluated with the pipeline presented in Section 3.2.

### 3.1. Illumination Sensor

**Figure 2:**
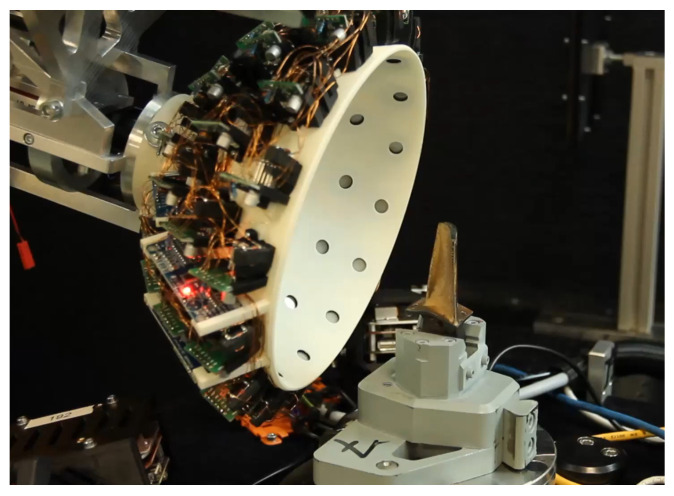
Prototype of the illumination sensor without outer shell mounted on an industrial robot.

**Figure 3:**
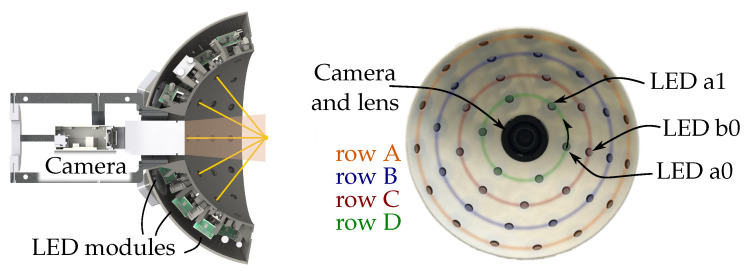
(**Left**): Cut through the CAD of the illumination sensor. All LEDs are aimed at the hemisphere centre. (**Right**): Inner hemisphere with LEDs with row and LED names.

The illumiation sensor is designed to realise multiple different lighting scenarios and capture the results with high resolution. For this, the prototype in Figure 2 was built. A total of 42 light-emitting diodes (LEDs) are arranged on a white hemisphere. A 12-megapixel camera (Allied Vision Manta G-1236, 4112 × 3008 pixels) is mounted in the middle of the surface and equipped with a 25 mm lens. The optical working distance of the sensor is in the range between 100 and 150 mm. The estimated field of view (FOV) is approximately 10–15 mm, while the estimates pixel resolution is 2–4 μm. The LED light sources are placed in multiple rows around the camera (cf. Figure 3).

Each LED is numbered according to the row and its position inside the row. Each row is colour-coded for visualisation purposes. The computer-aided design (CAD) (see Figure 3) shows a cut through the sensor highlighting the working distance of the camera and placements of the LEDs. The sphere diameter is designed so that the light sources point on the same spot. This spot resembles the optical working distance of the used lens.

**Figure 4:**
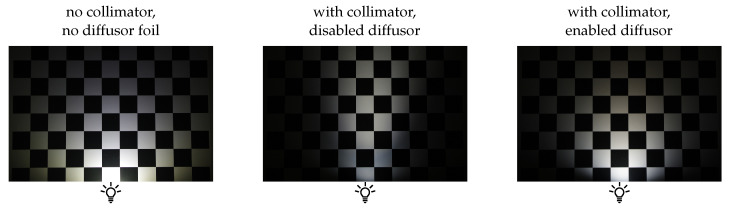
Comparison of the LED module of the illumination sensor with different configurations.

Each light unit has an LED driver to realise different currents and thus light intensities. Collimation lenses are used to reduce the radiation angles of the individual LEDs. In addition, a switchable diffusor foil is placed in front of the LED. Enabling it leads to a more diffuse light distribution and counteracts the effects of the collimation lens. The influence of the collimation lens and diffusor foil is shown in Figure 4.

Each unit can be enabled individually, which increases the number of possible lighting scenarios.

### 3.2. Reflection-Based Pixel Classification

In this section, the evaluation steps are described from data acquisition to segmentation of single pixels based on the surface reflectance properties.

#### 3.2.1. Data Acquisition

**Figure 5:**
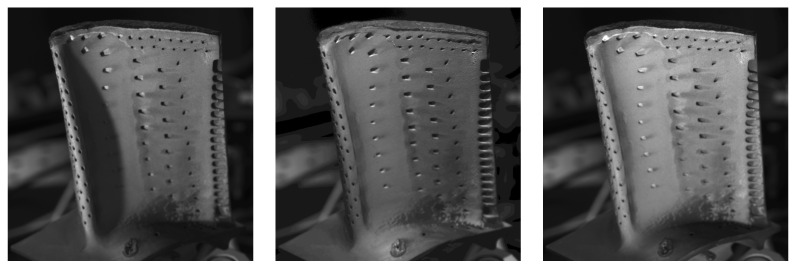
Example images with one enabled LED of the illumination sensor. (**Left**–**Right**): LED a4, b7 and c1. Images are brightened to increase visibility.

The data basis for the following steps is acquired by placing the sensor in its working distance and capturing a set of images with different lighting conditions. The set consists of an image with disabled LEDs, one with all LEDs enabled and 42 images with only a single LED enabled at a time with disabled diffusor. The first two images are used to derive a pixel mask for illuminated pixels, which will be used to reduce the number of pixels to be evaluated. Some example images of single LED images are shown in Figure 5.

**Figure 6:**
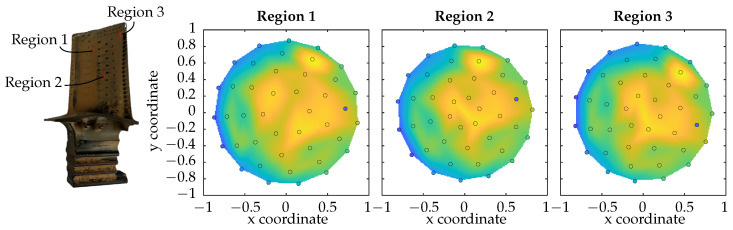
Scatterplots of light intensities for one exemplary pixel in the BRDF domain for 42 different lighting directions of the illumination sensor. Each plot corresponds to another region of the measured turbine blade. Cubic interpolation has been applied for values between data points.

Depending on the incident light direction, the pixel intensities vary from image to image. One reason is shadows which are cast due to the specimen’s shape. Another reason is the reflectance properties of the surface. The intensities for one pixel of different regions of the measured turbine blade seen in Figure 5 are shown in Figure 6.

Each dot represents the measured intensity of one pixel from each image in the image stack gathered during the measurement process. The *x*- and *y*-coordinates are derived from the normalised light vector. Values between the measurements have been interpolated cubically for visualisation purposes and are not used for further evaluation steps. While there are slight differences between each region, a common characteristic is the lower intensity for the outer LED rings A and B which increases with decreasing angle between surface normal and light direction in rings C and D. This behaviour is expected since a steeper angle between surface and incident light generally causes higher reflected intensities, whereas small angles lead to smaller values whilst keeping the view point unchanged. One data point can clearly be marked as an outlier. This was caused by a defect LED module which has a much lower light output. For the evaluation steps, this LED is excluded to avoid a negative influence on the results.

#### 3.2.2. Surface Normal Estimation

With the acquired image data, it is possible to determine the surface normals by applying a photometric stereo approach as presented in Section 2.2. Here, the pixel intensities of all images are used to get a overdetermined equation system. This can be solved in a least squares manner to estimate surface normals for each pixel.

#### 3.2.3. Reflectance Model Fitting

The estimated surface normals can further be used in the model fitting process. The required light vectors are taken from the CAD. Here, it is assumed that the light directions are equal for all pixels and the light is perfectly collimated in the working distance of the sensor. As shown in Figure 4, these assumptions deviate from the actual lighting conditions. Additionally, assembly-induced deviations can further influence the incident beam. Nevertheless, these simplifications seem feasible for this application as indicated by the presented results in Section 4.

To determine the view vectors, two approaches have been examined. One method assumes the view vectors to be parallel to the optical axis, thus assuming an orthographic camera model, and another approach applies the intrinsic parameters of a projective pinhole camera model [25,26], which was calibrated beforehand. Let

(7) x∈Z=0,…,w−1andy∈Z=0,…,h−1,

denote the pixel coordinates, where *w* and *h* are the width and height of the image. The orthographic approach defines the view vectors for each pixel of the image as follows:

(8) $${\vec{v}}_{x,y}=\left(\begin{array}{c} 0\\ 0\\ 1 \end{array}\right)\;\forall \;x,y.$$

Thus, the view vectors are the same for all pixels and point along the optical axis in positive *z*-direction.

The view vectors of the projective camera model are dependent on the intrinsic parameters of the camera, which are stored in the camera matrix C. For each pixel, the vectors can be calculated with the following equation,

(9) $${\vec{v}}_{x,y}=C^{-1}\left(\begin{array}{c} x\\ y\\ 1 \end{array}\right)\;,$$

which are then normalised to satisfy $∥{\vec{v}}_{x,y}∥=1$. The difference between both methods will be shown exemplarily in Section 4.1.

To determine the parameters of the anisotropic phong model (see Section 2.1), the common cosine weighted error function [27] is applied, minimising the error between calculated model intensities i and measured intensities $\hat{i}$, which are weighted with the cosine of the incident angle $\theta_{i}$. Thus, the error vector can be calculated as follows:

(10) $$e=\left(i-\hat{i}\right)\;\cdot \;\cos\theta_{i}.$$

**Figure 7:**
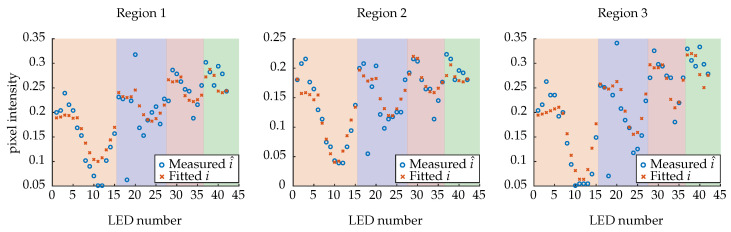
Fitted intensities vs. measured pixel intensities for the three regions. Sections are colour-coded to highlight the LED rows.

A Trust Region Reflective algorithm [28] is applied which minimises ∥e∥. Thus, the parameters $R_{s}$, $R_{d}$, $n_{x}$ and $n_{y}$ can be estimated. This process is performed for all selected pixels of the image, resulting in a M×4 parameter array, where *M* is the number of examined pixels. The fitted intensities for an exemplary pixel of different regions are shown in Figure 7.

As seen, the overall trend is modelled well with the chosen model and the identified parameter set. Since the fitting process is at risk of being ill-conditioned [22], the repeatability was tested. For this, the model was repeatedly fitted to the data using varying initial parameters. For this, the lower and upper bounds for $R_{s}$ and $R_{d}$ have been set to [0,1]. The allowed interval for $n_{x}$ and $n_{y}$ is set to [0, inf]. It has been observed that even with random starting values between 0.5 and 1.0, the optimiser generally converges to the same solution. However, because there is no ground truth, the overall validity of the model cannot be confirmed.

#### 3.2.4. Model Classification

Given the model parameters for each pixel, the aim is to distinguish groups of models with similar properties. Therefore, the available four parameters have been split into the part used to describe direction-dependent effects $n_{x}$ and $n_{y}$ and the parameters $R_{d}$ and $R_{s}$ which are used to model more diffuse or shiny material.

To assign these sets to different classes, a manual assignment process could be carried out, where a user selects multiple regions on the assumption of similar reflection properties. From this set of models, a “mean” model could be derived and used to assign other similar parameter sets. This process, however, is error-prone and strongly dependent on the user input.

**Figure 8:**
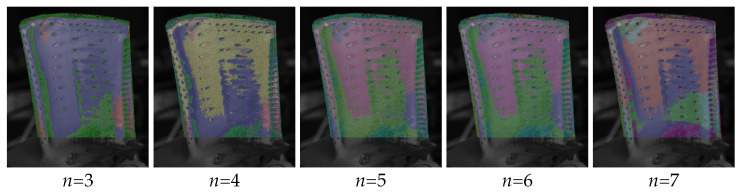
Influence of the specified number of classes for the classification results, starting from 3 classes left to 7 classes right.

Therefore, a K-Means [29] cluster algorithm was applied. Although this reduces the required user input, the number of expected classes *n* has to be specified, which affects the outcome of the classification. The influence of the number of classes is exemplarily shown in Figure 8.

While the “main” regions around the cooling holes are in the centre, the bottom right near the trailing edge and the area close to the top of the blade are already shaping with three classes, primarily close to the bottom of the blade, and on the edges, new classes are added.

It should be mentioned that these classification results are sensitive to the chosen region of interest and can vary depending on the observed surface area.

## 4. Results

**Figure 9:**
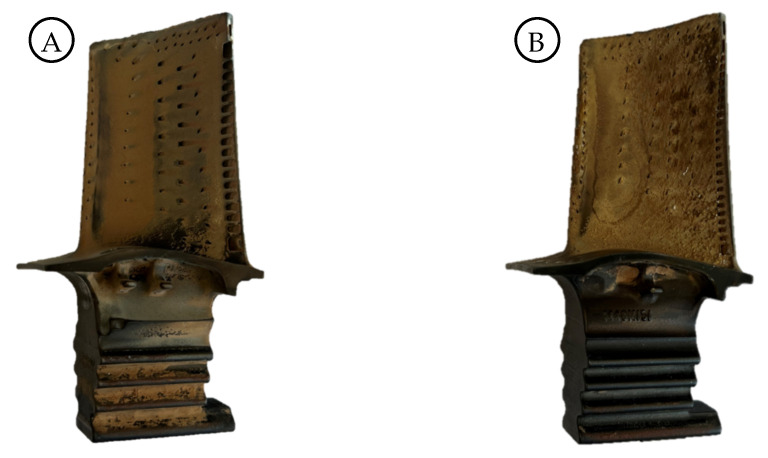
Turbine blades with different wear-related surface properties which were examined using the illumination sensor.

In the following section, measurement results of two different turbine blades (see Figure 9) are presented. In both cases, the pressure side was examined with the developed illumination sensor. The blades differ in their surface conditions: while blade A has a rather smooth surface with visible dark areas around the cooling holes, blade B shows significant surface structures with an apparently larger roughness.

Since it is assumed that the fitted reflectance model parameters are related to the surface roughness of the turbine blades, both blades have been examined with a confocal laser scanning microscope (Keyence VK-X200). Here, multiple surface measurements with 50 × magnification, a numerical aperture (NA) of 0.95 and a measuring field size of 300 μm× 200 μm have been carried out. The lateral resolution of the surface datasets is 0.3 μm. The results are shown as 3D plots and are supplemented with tables containing roughness parameters as defined in ISO 25178 [30]. The following parameters are included:

- Sa: Arithmetic mean height.
- Sz: Maximum (valley to peak) height.
- Sq: Root mean square height.
- Sp: Maximum height of peaks.
- Sv: Maximum height of valleys.
- Sku: Kurtosis of height distribution.
- Ssk: Skewness of height distribution.

In addition, the angular spectrum is calculated and compared to the directional parameters $n_{x}$ and $n_{y}$ from the fitted model. To visualise the dominating parameter, the difference is calculated as

(11) $$n_{diff}=n_{y}-n_{x}.$$

Since these parameters are not limited by any bounds, the largest absolute difference is set to 200. Thus, values closer to −200 represent a higher value for $n_{x}$, whereas 200 implies a bigger influence of $n_{y}$.

### 4.1. Turbine Blade A

**Figure 10:**
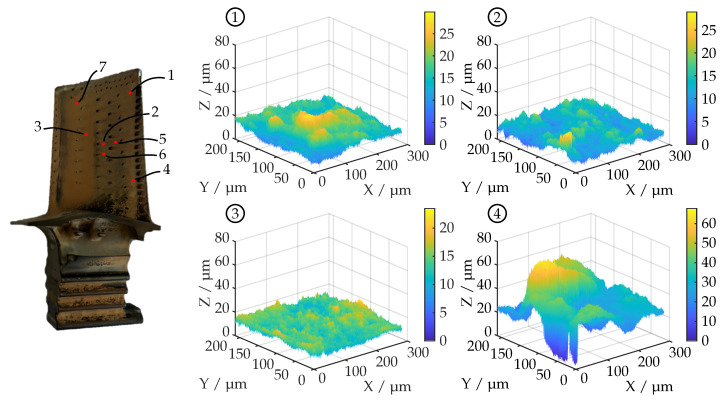
Four exemplary surface profiles on the pressure side of turbine blade A acquired using confocal laser scanning microscope (Keyence VK-X200, 50× magnification, NA = 0.95, 0.3 μm lateral resolution). The position of the respective measuring field is indicated by the numbers 1–4 in relation to the image of the turbine blade on the left-hand side.

Turbine blade A has a rather smooth surface with small rough sections (top right and bottom right). This is also shown in Figure 10 and Table 1, with area 4 deviating from the other roughness measurements. A total of seven surface measurements have been carried out.

**Figure 11:**
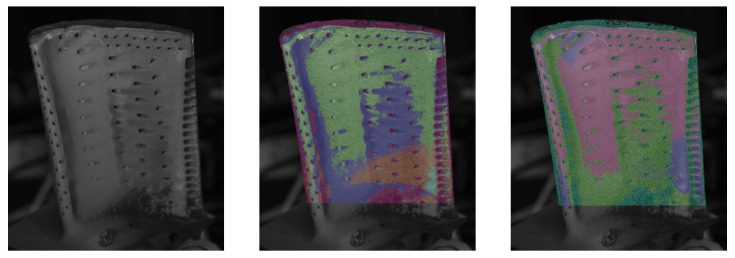
Result of K-Means clustering with 5 classes applied to the identified parameter sets of blade A. Each detected class is assigned a color. (**Left**): Reference greyscale image. (**Middle**): Orthographic camera model. (**Right**): Pinhole camera model.

The classification results based on the proposed approach are shown in Figure 11. Here, the models have been fitted using and orthographic and the pinhole camera model to determine the view vectors. It is to be expected that the influence of the chosen model increases closer to the border, since in this region, the difference between vectors is larger. As seen in both results, the angle has, in some areas, a significant influence on the result. Since the camera parameters are available and the simplification of the model seems to have an influence on the outcome, the projective model will be chosen as the preferred method for further investigations.

Comparing classification results based on the data acquired by the illumination sensor and the roughness parameters measured by the confocal laser scanning microscope, it can be seen that the parameters Sa, Sq and Sz do not show a clear relationship with the classes.

The calculated roughness parameters are shown in Table 1. The assigned row colours correspond to the colours assigned to the detected classes in Figure 11.

The kurtosis of the surfaces seems to follow the classification results, with *Sku* increasing from blue to red to green. The skewness also seems to match the different classes, with the smallest *Ssk* values in the red regions, an intermediate class in the blue regions, and the largest values in the green regions.

**Figure 12:**
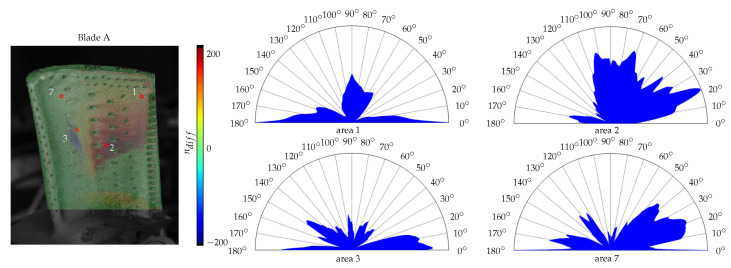
Difference of the directional parameters $n_{x}$ and $n_{y}$ and the angular spectra from the roughness measurements for blade A. The image on the left shows the location of the respective measuring points on the surface of the turbine blade under investigation.

The directional model parameters and the angular spectrum of the four roughness measurements for area 1, 2, 3 and 7 are shown in Figure 12. The texture orientation based of the angular spectrum varies. The spectrum in area 2 shows peaks towards 20° with additional ones at about 70° and 100°. This strong trend can also be found in the estimated model parameters. Area 3 and 7 are balanced with a slightly higher value for the *x*-direction. Area 1 shows peaks for 0° and 180° with another region expanding between 60° and 100°. While the other measurement areas mainly consist of similar colours, this region varies from pixel to pixel due to the surface structures. Thus, a precise assignment is not possible. Nevertheless, the results are not implausible.

An explanation for the discrepancies may be the different points of view of the sensor and microscope in combination with the complex geometry of the specimen. As a result, the directional dependency is estimated and analysed in two different coordinate systems. While the microscopy measurements were carried out orthogonal to the sample, the data of the illumination sensor were recorded with a larger incidence angle and for a significantly larger surface area.

### 4.2. Turbine Blade B

**Figure 13:**
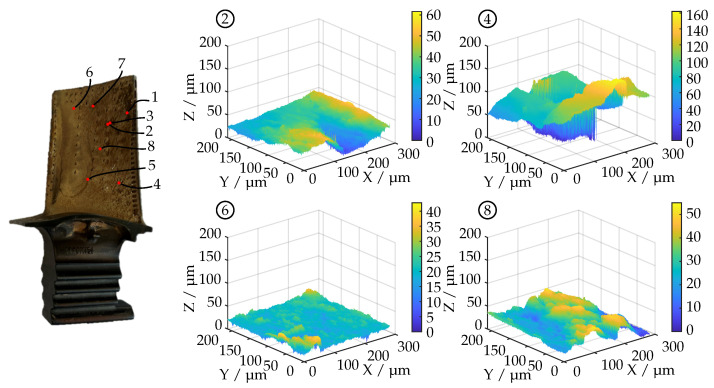
Four exemplary surface profiles on the pressure side of turbine blade B acquired using confocal laser scanning microscope (Keyence VK-X200, 50× magnification, NA = 0.95, 0.3 μm lateral resolution). The position of the respective measuring field is indicated by the numbers 2,4,6,8 in relation to the image of the turbine blade on the left-hand side.

This blade has a much rougher surface than blade A which is reflected by the surface measurements in Figure 13 and the parameters in Table 2. A visibly smoother surface area can be found in the curved area between leading edge and the cooling holes. For this specimen, a total of eight surface measurements have been carried out.

For this blade, areas 2, 4, 6 and 8 are shown exemplarily ranging from low-roughness surfaces in area 6 to more structured data with sharp edges in area 4.

The classification of the pixels was performed for five classes. The rows listed in Table 2 are coloured according to these results.

Similar to blade A, the different classes cannot be used to divide a specific range of the parameters Sa, Sq and Sz. These parameters suggest that area 3 and possibly area 2 should be in the same class as area 7 and 8, leaving area 1, 4 and 5 with higher measured roughnesses, with area 6 forming a class for low roughness.

A kurtosis-based classification does not seem to be feasible either. Here, area 2, 3 and 4 would be a better match with area 7 and 8 to form three classes without overlapping parameter ranges. Based on the skewness, the classification does not produce any overlapping value ranges. Negative values for *Ssk* belong to green regions, small values below 1.0 belong to the turquoise areas, and red to parameters above 1.0.

**Table 2:** Roughness parameters for blade B (row colours correspond to classification result in Figure 14 using the pinhole camera model).

Area	Sa (μm)	Sq (μm)	Sz (μm)	Sp (μm)	Sv (μm)	Sku	Ssk
1	9.10	12.36	81.60	23.22	58.37	5.03	−1.41
2	7.50	9.46	62.77	29.14	33.56	2.91	−0.51
3	8.94	10.89	58.76	24.61	34.14	2.55	−0.40
4	24.75	32.31	161.86	74.15	87.70	2.92	−0.43
5	10.00	13.40	89.23	30.43	58.79	4.33	−0.85
6	2.84	3.98	42.63	22.90	19.73	6.07	1.16
7	8.75	11.01	65.32	35.70	29.62	2.81	0.56
8	8.64	10.25	55.11	27.51	27.60	2.19	0.14

Figure 15 shows the difference of the directional parameters and the angular spectrum for blade B. This specimen is a lot more structured than blade A which results in a big range of $n_{diff}$ in a small region. Horizontal edges are more likely to be coloured blue, and vertical structures red. Smooth surface regions, like area 6, are in the middle of the available value range. The areas 3 and 8 are mainly red, which coincides with the angular spectrums. Area 1 shows similar characteristics to area 1 for blade A. Here, the region contains a bigger range of values for $n_{diff}$, which raises difficulties in assigning the correct object point. A notable discrepancy can be found for area 6. The angular spectrum suggests a much stronger vertical influence, which does not match the estimated directional parameters. This may be caused by the aspects stated for blade A regarding the unequal view points of both measurement systems.

## 5. Discussion and Further Work

The presented results show that a comprehensible relationship between identified reflection model parameters and the surface roughness parameters could not be fully confirmed nor declined. Based on the classification results of the fitted reflection models, it is not possible to define ranges for height parameters like *Sa*, *Sq* or *Sz*. However, there are indications of a relationship between skewness *Ssk* and kurtosis *Sku* in relation to the parameters of the reflection model for specific value ranges.

The investigation of the directional model parameters has shown that the estimation of directional structures based on the model parameters generally seems feasible. However, there are limitations induced by the geometry itself in combination with the view point of the sensor.

**Figure 14:**
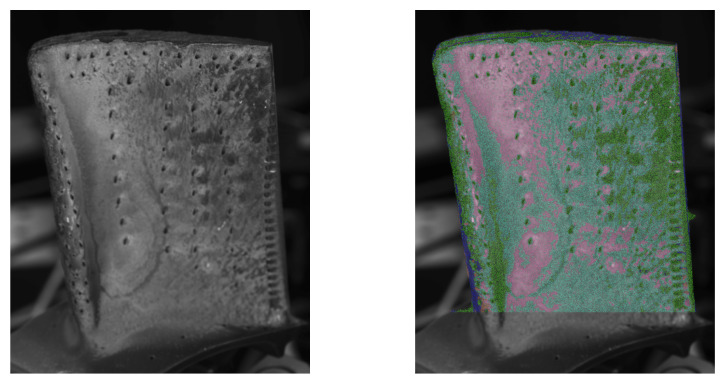
Result of K-Means clustering with 5 classes applied to the identified parameter sets of blade B.

**Figure 15:**
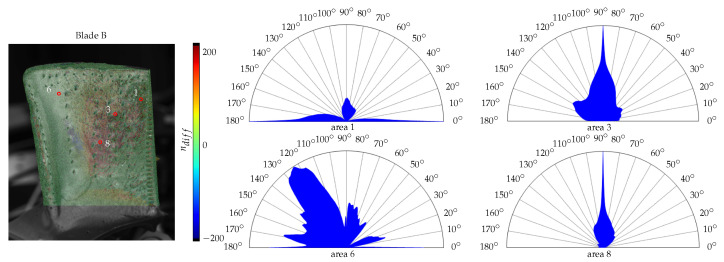
Difference of the directional parameters $n_{x}$ and $n_{y}$ and the angular spectra from the roughness measurements for blade B. The image on the left shows the location of the respective measuring points on the surface of the turbine blade under investigation.

Owing to the principle of the evaluation strategy which was pursued in this work, the estimated model parameters rely on the pixel intensities. These grey values are not only dependent on the roughness or the reflectance model, but can also be caused by a change of colour of the material, which in turn may be a sign of wear caused by, e.g., abrasion or adhesion. Affected areas may be put into a separate class as seen in the classification results from both blades (cf. Figure 11 and Figure 14), where darker surface patches have been segmented.

Nevertheless, there are multiple factors which can further influence the results. Roughness measurements and the surface assessment with the illumination sensor have been carried out on different systems. Thus, the assignment of measurement and the corresponding region on the blade have been done manually by relying on surface features like cooling holes or unique structures. In addition, only every tenth pixel of the illumination sensor data has been evaluated to reduce the computational effort. Thus, the pixels may not be matched with the correct measurement or vice versa.

Further influencing factors are the normal estimation process and the light vector approximation. Both directly affect the model fitting process. The former can be improved by applying more complex algorithms or by combining this sensor with 3D measurements systems to provide a more reliable surface orientation. The light direction model may be improved by assuming spot lights which, combined with the 3D information and a known sensor placement, result in individual light vectors for each pixel.

Another aspect is the influence of shadows caused by the macroscopic geometry of the specimen. During the model fitting process, all light vectors are considered to determine the model parameters. This may not hold true since some light directions are blocked by the blade. Given the 3D information of the blade, this effect can be taken into account by, e.g., calculating mesh intersections for each light ray and adjusting the data used accordingly.

The combination of 3D data and a predecessor of this sensor was shown by Melchert et al. [31]. Further research will be carried out in this direction to assess the influence of the aforementioned aspects.

**Figure 16:**
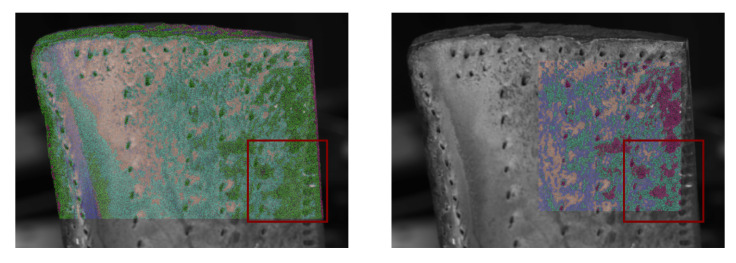
Comparison of clustering results for the same parameters but different selected sections. With a smaller evaluated surface area (**right**), more classes occur in the highlighted region than with a larger evaluated surface area (**left**).

Another factor is the classification step of the identified models. The region of interest has been set manually to exclude non-relevant areas like the turbine blades’ platform or background. However, this influences the results of the classification step since more parameter sets have to be divided into the same number of clusters. This effect is shown in Figure 16. In the highlighted region, the reduction in the area leads to more classes with deviating sizes.

Applying different cluster algorithms like DBSCAN [32] or OPTICS [33], which do not require the a priori information about the number of clusters, was not successful. The surface is evenly worn, leading to equally evenly distributed model parameters, which cannot be separated with the aforementioned algorithms.

## 6. Conclusions

In this work, an LED- and camera-based illumination sensor was developed for reflection-based characterisation of turbine blade surfaces. The system uses a hemispherical 42-LED arrangement and a high-resolution camera to acquire multi-angle image data, from which an anisotropic BRDF model is fitted on a per-pixel basis. This enables a reflectance-based segmentation of technical surfaces into regions with similar optical behaviour. Two turbine blades with contrasting surface conditions (comparatively smooth vs. visibly rough) were examined. Independently measured roughness parameters (*Sa*, *Sq*, *Sz*, *Ssk*, *Sku*) from confocal laser scanning microscopy served as reference data. While not consistent, a strong relationship was observed between BRDF parameters and height-based roughness metrics (*Sa*, *Sq*, *Sz*), and the results indicate that distribution parameters (*Ssk*, *Sku*) and directional BRDF parameters show clearer qualitative trends and are able to capture texture orientation and surface condition differences. However, based on the current dataset, coincidental agreement between BRDF parameters and roughness descriptors cannot be ruled out completly. As a result, we suggest a working hypothesis, for future investigations, that there is a correlation between the distribution parameters *Ssk* and *Ssu*, and the BRDF parameters.

Several aspects of this work are novel. First, anisotropic BRDF modelling, well established in computer graphics, is applied here to complex industrial turbine blade geometries with spatially varying wear, rather than to idealised shapes such as spheres, cylinders, or flat samples. Second, the combination of multi-angle photometric imaging using a 42-LED hemisphere with photometric stereo and BRDF fitting forms a practical pipeline for industrial surfaces that is more suitable for complex geometries than traditional gonioreflectometers. Third, the study provides initial qualitative evidence that statistical roughness properties—especially distribution parameters such as skewness and kurtosis—may exhibit relationships with BRDF parameters, suggesting that reflectance encodes aspects of surface texture beyond pure height information. These indications, however, require systematic quantitative investigation in future work.

From an application perspective, the developed sensor and methodology are particularly relevant for turbine blade inspection in maintenance, repair, and overhaul (MRO) scenarios. Here, the system can support rapid, wide-area surface condition assessment after operation and guide technicians to regions that warrant more detailed, time-intensive measurements with confocal or tactile instruments. This has the potential to reduce inspection time from hours, for full-surface high-resolution scanning, to minutes by enabling a triage workflow: the illumination sensor identifies areas with wear, erosion, oxidation or surface damage, which are then investigated in detail using established roughness measurement systems. In quality assurance (QA) during manufacturing, the approach can be used for post-production inspection of turbine blades to detect defects such as tool marks or insufficient finishing across the entire surface, improving confidence that sampled regions are representative before release.

Beyond these direct industrial applications, the methodology offers a basis for research into the relationship between surface reflectance and functional properties such as aerodynamic efficiency, heat transfer behaviour, and wear mechanisms. By linking BRDF parameters with statistical surface descriptors and texture orientation, the approach can contribute to optimising surface finishes for specific performance objectives. Future work will extend validation to other component geometries, perform comprehensive quantitative analysis of BRDF–roughness relationships, and integrate the illumination sensor into broader condition-based maintenance and digital inspection pipelines.

## Figures and Tables

**Table 1:** Roughness parameters for blade A (row colours correspond to classification result in Figure 11 using the pinhole camera model).

Area	Sa (μm)	Sq (μm)	Sz (μm)	Sp (μm)	Sv (μm)	Sku	Ssk
1	2.89	3.69	30.07	14.00	16.06	3.08	0.39
2	1.97	2.49	28.96	17.37	11.58	3.80	0.67
3	1.46	1.84	23.60	10.72	12.87	3.28	0.11
4	8.45	10.34	67.95	34.97	32.97	2.98	0.54
5	1.46	1.93	22.43	10.35	12.07	4.33	0.64
6	2.13	2.72	25.16	13.76	11.40	3.73	0.75
7	1.36	1.73	18.08	9.11	8.96	3.32	0.20

## Data Availability

Data underlying the results presented in this paper are not publicly available at this time but maybe obtained from the author upon reasonable request.

## Abbreviations

The following abbreviations are used in this manuscript:

BRDF	Bidirectional reflectance distribution function
PS	Photometric stereo
LED	Light-emitting diode
MRO	Maintenance, repair and overhaul
